# Design and implementation of a large and complex trial in emergency medical services

**DOI:** 10.1186/s13063-019-3203-0

**Published:** 2019-02-08

**Authors:** Maria J. Robinson, Jodi Taylor, Stephen J. Brett, Jerry P. Nolan, Matthew Thomas, Barnaby C. Reeves, Chris A. Rogers, Sarah Voss, Madeleine Clout, Jonathan R. Benger, In collaboration with the AIRWAYS-2 study team

**Affiliations:** 1 South Western Ambulance Service NHS Foundation Trust, Exeter, UK; 2 Clinical Trials and Evaluation Unit, Bristol Medical School, University of Bristol, Bristol, UK; 3 Centre for Perioperative Medicine and Critical Care Research, Imperial College Healthcare NHS Trust, London, UK; 4 Department of Anaesthesia, Royal United Hospitals Bath NHS Foundation Trust, Bath, UK; 5 University Hospitals Bristol NHS Foundation Trust, Bristol, UK; 6 Faculty of Health and Applied Sciences, Glenside Campus, University of the West of England, Bristol, BS16 1DD UK

**Keywords:** Out-of-hospital cardiac arrest, Cardiopulmonary resuscitation, Emergency medical services, Research design

## Abstract

**Background:**

The research study titled “Cluster randomised trial of the clinical and cost effectiveness of the i-gel supraglottic airway device versus tracheal intubation in the initial airway management of out-of-hospital cardiac arrest (AIRWAYS-2)” is a large-scale study being run in the English emergency medical (ambulance) services (EMS). It compares two airway management strategies (tracheal intubation and the i-gel) in out-of-hospital cardiac arrest. We describe the methods used to minimise bias and the challenges associated with the set-up, enrolment, and follow-up that were addressed.

**Methods:**

AIRWAYS-2 enrols adults without capacity when there is no opportunity to seek prior consent and when the intervention must be delivered immediately. We therefore adopted a cluster randomised design where the unit of randomisation is the individual EMS provider (paramedic). However, because paramedics could not be blinded to the intervention, it was necessary to automatically enrol all eligible patients in the study to avoid bias. Effective implementation required engagement with four large EMS and 95 receiving hospitals. Very high levels of data capture were required to ensure study integrity, and this necessitated collaborative working across multiple organisations. We sought to manage these processes by using a large and comprehensive electronic study database, implementing efficient trial procedures and comprehensive training.

**Results:**

Successful implementation of the study design was facilitated by the approaches used. The necessary regulatory and ethical approvals to conduct the study were secured, and benefited from strong patient and public involvement. Early and continued consultation with decision makers within the four participating EMS resulted in a coordinated approach to study set-up. All receiving hospitals gave approval and agreed to collect data. A comprehensive database and programme of training and support were implemented. More than 1500 paramedics have been recruited to the study, and patient enrolment and follow-up has proceeded as planned.

**Conclusion:**

Care provided by EMS needs to be based on evidence. Although participants may be experiencing life-threatening emergencies, high-quality pre-hospital research is possible in well-designed and well-managed studies. The approaches described here can be used to support successful research that will lead to improved treatment and outcomes in similar patient groups.

**Trial registration:**

ISRCTN08256118. Registered on 22 July 2014.

**Electronic supplementary material:**

The online version of this article (10.1186/s13063-019-3203-0) contains supplementary material, which is available to authorized users.

## Background

The research study titled “Cluster randomised trial of the clinical and cost effectiveness of the i-gel supraglottic airway device versus tracheal intubation in the initial airway management of out-of-hospital cardiac arrest (AIRWAYS-2)” is a randomised controlled trial of an intervention performed by emergency medical (ambulance) service (EMS) providers in the pre-hospital setting in England [1, 2]. The study aims to determine whether a new supraglottic airway device (the i-gel) is superior to tracheal intubation when used as the first advanced airway device in adult patients who have sustained a non-traumatic out-of-hospital cardiac arrest (OHCA). It is funded by the National Institute for Health Research Health Technology Assessment Programme (HTA reference 12/167/102).

When a cardiac arrest occurs, the heartbeat and breathing cease and the patient becomes unconscious immediately [3]; this means that there is no opportunity to obtain prior consent. In addition, the outcome from cardiac arrest is very poor, and more than 90% of patients do not survive to hospital discharge [4]. OHCA is sudden and unpredictable and conducting ethical research in patients who have suffered a cardiac arrest and are immediately incapacitated is challenging. Relatively small gains in survival of 3–4% would be clinically meaningful and worthwhile [5], providing the intervention is cost effective. This means that large sample sizes are necessary, and missing data could substantially undermine the validity of study results. To improve survival following OHCA, high-quality research is required; however, as described, the logistical and ethical challenges of conducting research in this patient population are challenging. The pre-hospital phase of care is only part of the patient pathway in OHCA. Hospital teams play a key role in data collection and patient follow-up, and effective engagement with receiving hospitals is therefore necessary. Very few pre-hospital studies are randomised controlled trials (RCTs) [6], and some previous pre-hospital RCTs have proved unsuccessful [7]. Appropriate study features are therefore required to ensure that research in this patient population is:

- Ethical, socially acceptable, and compliant with legal and regulatory requirements
- Practicable in an emergency setting where patient care is the priority
- Of high methodological quality, addressing the unique challenges of this environment
- Implemented successfully to answer the study question

The AIRWAYS-2 study protocol and primary results have been published previously [1, 2]. This paper outlines the key methodological design features of AIRWAYS-2, and describes how these facilitated successful implementation of the study in receiving hospitals to address the requirements outlined above. Pre-hospital issues will be described in a separate publication. Our study design choices and experiences may inform future similar studies in patients suddenly incapacitated by life-threatening emergencies, to enhance research quality and success, and improve long-term outcomes for patients.

## Methods: study design

### Cluster randomisation and automatic enrolment

In AIRWAYS-2, the potential participants are unconscious, in need of immediate emergency care, and clinical necessity is therefore the overriding priority. For this reason, it was not possible to design the study to randomise individual patients, and a cluster randomised design was deemed most appropriate. We chose to randomise the EMS provider (paramedic), treating each participating paramedic as a cluster. This choice meant the study had a large number of clusters, with average cluster size being relatively small (the median number of OHCAs attended by a paramedic annually was three in our previous feasibility study [8]), which minimises the loss of efficiency due to observations within clusters not being independent.

Paramedics were randomised in a 1:1 ratio using a purpose-designed, secure internet-based system. This ensured that the number of paramedics in each group was equal; however, some imbalance in the number of patients enrolled was possible as a result of chance. Since it was not possible to blind the participating paramedics to the study intervention, it was necessary to ensure that all eligible patients were enrolled in order to avoid bias. We therefore adopted a model whereby every eligible patient attended by a participating paramedic was automatically enrolled into the study. In this way, the participating paramedics could not influence whether a patient was enrolled, while EMS control room staff, who allocate paramedics to individual patient calls, were blinded to both paramedic participation and allocation. Where two participating paramedics were allocated to the same patient call, the first paramedic ‘at the patient’s side’ was assigned; if both paramedics arrived together, the attendant paramedic, rather than the driver was included for study purposes. Patients could either be consciously enrolled by the participating paramedic, or automatically enrolled following identification of their eligibility subsequent to the OHCA. Data for OHCA are routinely collected and reported by ambulance services [9] and interrogation of these, in addition to the study-specific data collection, meant that all (i.e. as close to all as it is possible to be sure of) eligible participants were included; we do not anticipate that there was a significant level of ‘missingness’ in the data.

### Data collection, patient information and consent

Research in OHCA is challenging because it requires the recruitment of incapacitated adults without the opportunity to obtain prior informed consent. Furthermore, because AIRWAYS-2 is a trial of advanced airway management in the very early stages of OHCA, the intervention is completed within a very short time from the onset of the cardiac arrest and before consent can be sought. For this reason, we had to demonstrate that strict ethical safeguards and robust patient and public involvement were in place when we designed the study and that there was collective equipoise between the two study treatments.

The following features addressed these challenges:

- We decided to use a model of deferred consent for survivors, and a waiver of consent for those who did not survive to hospital discharge. We therefore needed to obtain approval from the Confidentiality Advisory Group (CAG) in England to be able to retain data on our primary outcome (modified Rankin Scale score at hospital discharge or 30 days post-OHCA, which assigns a score of 6 to non-survivors) for all participants enrolled in the study.
- We considered a model of approaching relatives to seek their agreement to retain the data collected from patients who did not survive; however, there is no basis for this option in English law. There is also a risk that it could generate differential consent rates for patients who did and did not survive, undermining the internal validity of the study, which requires complete or near-complete primary outcome data on all patients enrolled. Furthermore, we are aware that informing relatives that their recently deceased loved one was involved in a research study has a high risk of increasing distress and uncertainty without benefit [10–12]. Therefore, after careful consideration, and with the agreement of our patient public involvement (PPI) group, we decided not to inform the relatives of enrolled patients who did not survive the initial cardiac arrest that they had participated in a research study. This approach was possible because both airway interventions are currently in use in English EMS, and so OHCA patients could have received one intervention or the other during routine patient care outside the study.
- For participants who survived to hospital discharge, we sought retrospective consent to collect follow-up data at 3 and 6 months. Our PPI group recommended that patients be informed of the study and asked to consent at the time that they were discharged from the intensive care or coronary care unit, but before hospital discharge. Seeking consent at this time required hospital-based research nurses to carefully monitor the status and location of patients recovering from OHCA, which was time consuming and also led to some patients being discharged from hospital before they could be approached. Therefore, to ensure all participants could be informed about the study, we also obtained approval to request written consent via post.

To maximise consent rates, participants were given three consent options:

1. Active follow-up. Data were collected from their medical records and they were invited to complete questionnaires about their ongoing health and wellbeing at 3 and 6 months after OHCA.
2. Passive follow-up. Data were collected from the patient’s medical records, but they were not contacted again, nor invited to complete follow-up questionnaires.
3. No further involvement. No further information was collected, but stating clearly that the information already collected would be retained and included in the data analysis. Anonymity of the participant was assured.

A proportion of participants who experience an OHCA remain incapacitated, and so the study was designed so that a personal consultee (usually a close relative) could provide an opinion as to the follow-up option that would likely be preferred by the patient.

### Securing regulatory approvals

At the time of set-up, the approval process for research involving patients at hospitals in the UK involved each hospital completing a site-specific information (SSI) form, detailing the research activities performed at that site [13]. Hospitals were then required to provide local research governance permissions, which included signing a contract [14].

We recognised that obtaining approval from each hospital would be time consuming, and a streamlined approach was required. Table 1 provides details of the process. The key objective was to reduce the burden of obtaining approvals.

**Table 1:** Streamlining the hospital approval process

Normal process	AIRWAYS-2	Rationale for streamlining	Advantage for hospital
Site-specific assessment form for each hospital	Generic site-specific form for all hospitals		No need for hospital to complete information about local site activities
Patient consent and information forms on local hospital trust headed paper	Patient consent and information forms on sponsor/trust headed paper	Patient recruitment being carried out by ambulance services. No change of practice in hospital and activity limited to follow-up	No need for hospital to spend time localising patient documents, as documents provided to hospital by study team on ambulance trust/sponsor headed paper
Principal Investigator	Local collaborator	No serious adverse events expected to occur in hospital; likely to occur at roadside and would be reported by ambulance trust	A research nurse, rather than a doctor, could act as the hospital’s main point of contact for the study
Third-party contract requiring sign-off at each hospital	Simple one-page document ‘statement of responsibilities’ issued to hospital sites; no signature required	Hospital not responsible for participant enrolment or delivering intervention so a simpler agreement is sufficient	No complicated legal terminology for contracts department to review. No signature, therefore less administration for research and development teams

The lead Clinical Research Network (CRN) offered extensive support during the study set-up phase and agreed that a generic SSI form could be used to gain hospital approvals. This meant that all hospitals could be provided with the pre-populated generic SSI form, reducing the administrative burden. Obtaining contract signatures was identified as an aspect of the approval process that had the potential to cause significant delays [13]. To mitigate this, we provided sites with a ‘Statement of Responsibilities’ document in place of the usual contract [14], which detailed the support that each hospital would receive in respect of their participation. This document did not require individual site sign-off.

As part of the approval process, it is usual for hospitals to format study information and consent forms on their own headed paper. Given that the study interventions were delivered in the pre-hospital phase of care and the in-hospital activities related to follow-up data collection, this was not necessary and standard documents could be provided, further reducing administration locally.

## Methods: study implementation

### Identification and engagement of key stakeholders

All enrolled patients transferred to the emergency department required follow-up data to be collected in hospital. Hospitals identified as potential receiving sites were any of those within, or bordering the geographical area served by, the four participating EMS. It was not possible to predict or influence which hospital an enrolled patient would be taken to, and this meant that all 95 hospitals served by the four ambulance services needed to participate in the trial. If a hospital refused to take part or could not provide the necessary approval, we could not collect data for enrolled patients taken to that hospital.

To facilitate engagement from all hospitals it was agreed that a multi-faceted approach would be required. We planned to identify key stakeholders who could promote awareness of the study and facilitate communication. Early engagement and good communication with hospitals was essential, as was a clear outline of what their participation would involve. The design of the study, with the intervention administered solely during the pre-hospital phase of care and hospital research activities being limited to obtaining informed consent and data collection meant that Principal Investigators (PIs) were not required at hospital sites; rather a non-medical local collaborator (LC) could be identified as the main point of contact. In the UK, the usual approach is for hospitals to be invited to express an interest in taking part in a research study, and for decisions regarding participation to be made according to local processes [15]. A different approach was required to ensure that all hospitals that might receive an AIRWAYS-2 patient took part. In our communications, we provided concise study information and a clear rationale for including all receiving hospitals, rather than inviting hospitals to express an interest in participating.

### Training and management of research staff at a large number of hospitals

Another major challenge was training the staff at the receiving hospitals. We needed to train over 300 staff at 95 hospitals. When setting up a study it is imperative to ensure the research staff have the appropriate qualifications and training and understand the study protocol. Training is often delivered in the form of face-to-face trial initiation visits at each of the research sites. This can significantly increase the set-up costs and delay opening a site. To mitigate this, we decided to train the hospital staff using online training videos and a detailed, online study manual; this would enable staff to revisit the material as often as they wished and would also make it easier for new staff to join the hospital study teams.

### Design and delivery of a comprehensive electronic study database

**Fig. 1:**
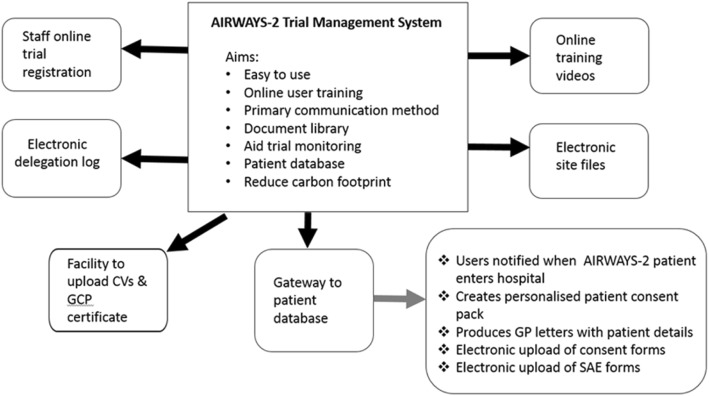
Electronic trial management system. CV Curriculum Vitae, GCP Good Clinical Practice, GP general practitioner, SAE severe adverse event

We also invested resources in developing an in-house, bespoke electronic management system that would help manage hospital-specific documentation and facilitate access to central study documents. Figure 1 shows the different components of the electronic management and training system.

In designing the electronic management system there were several objectives in mind. The system had to function as the primary communication tool between the study team and the hospital staff, facilitate online training, provide a document library, be easy to use, and reduce the carbon footprint of the trial.

The system enabled users (principally research staff responsible for data collection in the pre-hospital and in-hospital phases of care) to register for a study account online, complete an electronic delegation log, and upload relevant information and certificates. It was designed to allow users to select their delegated tasks (as appropriate to their role within the study and differing for pre-hospital and in-hospital staff) and for approval for access to be granted by the study manager once the tasks and documentation checks were verified as accurate and complete. All sites had access to the trial master file that was maintained by the study team and a local site file that enabled sites to upload site-specific documents.

The trial management system automatically generated a unique study identifier and produced patient-specific document packs once a minimum dataset had been uploaded by the research paramedics. Depending on the consent option selected by the patient or their consultee, the trial system created a set of electronic case report forms (eCRF; a copy of the eCRF is available in Additional file 1) to enable the correct data to be collected for each individual patient. This helped to reduce the workload for the sites, as contact information (for patients and regional research teams) was automatically pre-filled and the correct forms printed. The database had inbuilt and complex data verification and enabled data queries to be sent to research paramedics on a weekly basis.

## Results

We obtained study-wide research ethics committee approval in September 2014, with support for automatic enrolment of eligible patients. Our PPI group provided valuable advice which guided various aspects of the study design; we described their input in our applications to demonstrate their role in addressing the major ethical challenges. At the same time, we obtained approval from the CAG to collect primary outcome data without prior consent.

Four EMS agreed to collaborate in the study. Involving key stakeholders from the ambulance services at an early stage of the planning process enabled the identification of potential issues so that differences between services could be addressed. At two investigator meetings held during set-up, study-wide decisions on clinical practice, logistical issues, and study management were made. We agreed a common communications strategy that addressed paramedics’ concerns, and enabled the wider community (potential patients, EMS providers who were not participating, and healthcare providers) to be informed about the study. We commenced the approval process for the four EMS on 27 January 2015 and the NHS permissions were in place by 5 March 2015.

**Fig. 2:**
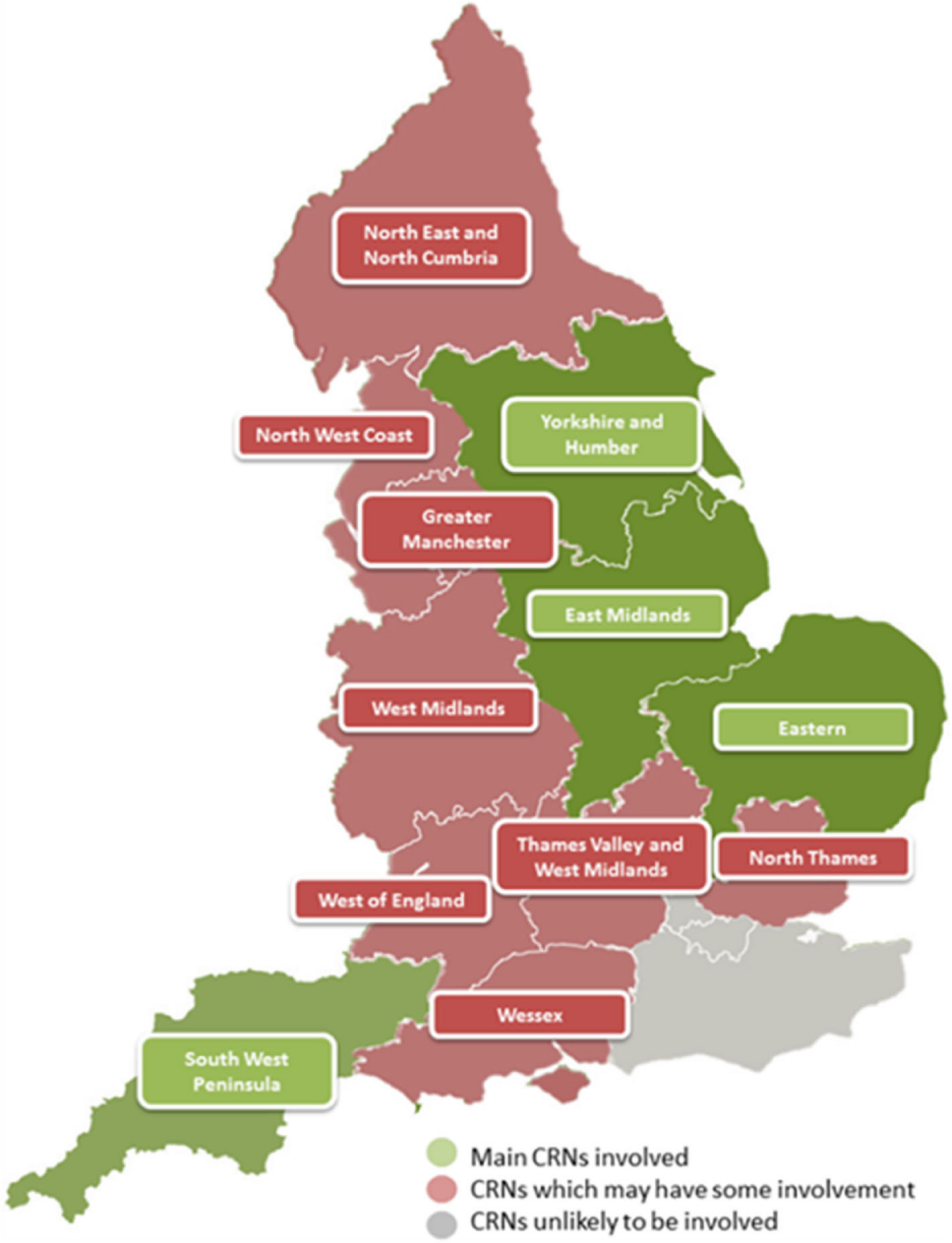
Clinical Research Network (CRN) involvement in the AIRWAYS-2 trial

We mapped the local National Institute for Health Research Clinical Research Networks (NIHR CRNs) [16] (Fig. 2) covering the study sites, based on the destination hospital to which an AIRWAYS-2 patient might be taken.

The lead CRN publicised the trial in their e-newsletter for the research community, and facilitated discussions with the critical care specialty research leads in the relevant CRN regions. This enabled the specialty leads to be approached for help in setting up the study in their local hospitals. A teleconference to further engage with the speciality leads was held and proved fruitful; they provided insights regarding variations in processes across the different hospital sites, gave valuable advice on how to utilise resources in each of their regions, agreed to promote the study at local meetings, and helped to identify suitable contacts within each hospital. Six of the 11 invited specialty leads and representatives from three of the four main CRN regions that covered the ambulance services took part in the teleconference. The representatives from the three CRNs were able to highlight any potential issues with study support in terms of lack of dedicated research nurses at individual sites. This enabled us to plan effectively and we identified hospitals that would potentially need the study regional research nurses to assist with data collection.

We used the CRN mapping (previously described) to identify LCs during the planning phase. By identifying LCs early on, the hospital teams had time to discuss how the study would be implemented locally, and were able to nominate clinical staff in both emergency and critical care departments who would assist in tracking patients and collecting the required data. With the support of the critical care speciality research leads and the local CRNs, 61 of the 95 LCs were identified before hospital sites were invited to take part in the study. Most of the hospitals accepted that the role of LC could be undertaken by non-medic staff (research nurses in the majority of cases). Four hospitals requested that a doctor undertake this role, and in these sites an intensive care consultant was identified. The identification of LCs before opening the study facilitated internal discussions, raised awareness of AIRWAYS-2, and paved the way for the opening of the study.

Obtaining NHS approvals was facilitated by the approaches used. We formally initiated the approval process for hospitals on 14 April 2015, and received the first approval 6 days later. Ninety-two of the 95 hospitals accepted the ‘Statement of Responsibilities’, with only three sites requesting a formal contract. We went on to receive NHS management permission from all 95 hospitals identified as potential receiving sites, with the last site agreeing to participate on 4 November 2015.

**Fig. 3:**
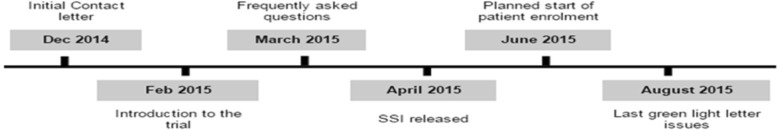
Timeline for hospital communications. Early correspondence with hospitals took the form of a trial introduction letter which answered the question “Why do Acute Trusts need to know about this trial”. Follow-up correspondence was sent 2 months later; this provided a trial design summary and information about the incentives the hospital would receive if they admitted an AIRWAYS-2 participant. Further correspondence was sent 1 month later; this provided targeted information answering common questions received from the hospitals. SSI site-specific information

Communications with hospital staff commenced 6 months before the planned start of patient enrolment. Figure 3 shows the timeline for communication.

The use of an electronic management system greatly enhanced the efficiency of the study; staff were able to access the electronic resources in their own time which meant that face-to-face site initiation visits were unnecessary and that the study manager was able to conduct these by telephone. In addition, the study team were able to monitor training records and site files without visiting the local hospital. The resources required to manage study amendments were reduced significantly; new study documents could be uploaded to the trial master file and all sites were then able to access these immediately.

Hospital research staff were initially uncertain about online training and electronic site files, but after receiving the training manual and using the electronic management system, the feedback reported to the regional research nurses was extremely positive. One of the most important benefits of the electronic management system was its role as an efficient primary communication tool. On enrolment of an eligible patient, a minimum dataset was entered manually by the research paramedic (these data were not linked to hospital clinical records or other routine data sources); the system then allowed the central research team to notify hospital staff via email that an AIRWAYS-2 patient had entered their hospital, and enabled staff to track the patient through their hospital pathway, approach the patient for deferred consent at the correct time, and enter their data directly onto the database. In some auto-enrolment cases, some months had elapsed between the OHCA and data entry and this meant that the patient was no longer in hospital; these patients were sent a postal invitation by the hospital staff.

Patient recruitment to the AIRWAYS-2 study commenced on 1 June 2015. More than 1520 paramedics were recruited, randomised, and trained across the four collaborating EMS. Patient recruitment was open for just over 2 years and closed on 13 August 2017. The primary clinical results have been reported previously [2].

The key lessons learnt from the design and implementation of this study are summarised in Table 2.

**Table 2:** Key lessons from the AIRWAYS-2 trial

• When the randomisation of individual patients is not possible, a cluster randomised design may be considered. A large number of small clusters will reduce intra-class correlation.	
• Automatic enrolment of all eligible patients minimises the risk of bias in circumstances where those responsible for participant enrolment are not blinded to treatment allocation.	
• For research in emergency situations it may be necessary to establish ways of collecting outcome data that do not rely on patient consent. In England, approval from the Confidentiality Advisory Group (CAG) can facilitate this.	
• Strong patient and public involvement (PPI) in study design helps to address challenging ethical and patient consent issues.	
• Hospital teams may not be used to collaborate in emergency medical (ambulance) services-led research.	
• Once an emergency medical (ambulance) service begins patient recruitment, any of the hospitals in the area covered by that service could potentially receive a patient. Therefore, a phased approach to opening hospital sites is not possible and alternative approaches are required.	
• Early identification of key stakeholders at participating healthcare organisations, and the provision of concise study documents, facilitates effective engagement and raises awareness of the study.	
• A study-wide communications strategy, approved by an ethics committee, enables local communities and healthcare organisations to be informed about the study, and minimises the risk of unwilling patients being enrolled.	
• An electronic system can support effective and efficient study management; it facilitates online training for site staff, provides an up-to-date document repository, and enables printing of patient-specific document packs.	
• For patients recruited to clinical trials in emergency situations a flexible approach to follow-up will maximise participation.	

## Discussion

AIRWAYS-2 is a large and complex study that aims to guide future initial airway management by EMS providers (paramedics) in patients experiencing out-of-hospital cardiac arrest. All patients enrolled in the trial lacked the capacity to consent, and the time-critical nature of the condition meant that there was no opportunity to identify or approach a consultee. Research in the emergency setting is challenging but essential if we are to improve future clinical practice [17]. The design of AIRWAYS-2 needed to be acceptable to patients, their family and carers, with due consideration of the many ethical issues that the study raised. We were guided by our patient and public group in this regard.

Blinding of individual paramedics was clearly not possible; this presented another challenge because it required that all patients attended by participating paramedics were included. The only way to achieve this was enrol eligible patients automatically. To enable this, we needed to gain approval for the collection and retention of a minimal dataset without consent. Legal provisions enable research in an emergency setting providing all proposed activities have approval from an appropriate research ethics committee [18]. The ethics committee demonstrated a high degree of understanding of the nature of prehospital research; recent increases in research activity by EMS and previous work completed by our team are likely to have been beneficial in this regard [7].

The set-up of AIRWAYS-2 was a large undertaking, and we identified a widespread lack of familiarity with the unique features and complexity of pre-hospital research. Many hospitals had no experience of participating in EMS-led studies, or of approaching patients for consent who had been enrolled in a study outside of hospital. There was a lack of understanding that the automatic enrolment of patients by participating EMS providers meant that hospitals might receive a recruited patient at any point following activation of the ambulance service site, and that the patient was already enrolled in the study under a waiver of consent. Consequently, it was not possible for us to take a staggered approach to set-up. We recognised the potential risks that delays in obtaining approvals would pose to the successful implementation of the study [13, 19] and we adopted a proactive approach to managing these. Early and effective engagement and communication with hospital clinical and research management staff, regional clinical leads, and research network colleagues were key factors in this. In regions where we were unable to engage with the CRN representative or specialty leads it was necessary to follow-up with hospital teams individually, and the workload and the time taken to engage with the appropriate teams was increased in these cases.

One of the greatest risks to the study was the requirement for all hospitals to agree to follow-up participants, and we recognised that it would be difficult to achieve approval from all hospitals that might receive an enrolled patient [20–22]. At the time of set-up, hospitals were assessed on various performance metrics as part of the national research governance process; one of these metrics was the time taken to review and provide approval for studies. In 16 of the 95 hospitals, it was not possible to identify an LC before the start of patient recruitment. In these sites, the hospital risked a metric target not being met, and seven hospitals initially abandoned the study as a result. We adopted a flexible approach, making allowances for the differing models, challenges, and staff shortages within individual organisations in order to engage all hospitals successfully. When a dedicated research nurse was not available in an individual hospital, we agreed that the AIRWAYS-2 regional research nurses would complete follow-up for admitted patients. The majority of hospitals had agreed to support the study in time for patient recruitment to commence. In sites where we had not received approval within 5 months, the sponsor escalated the issue to senior directors within the hospitals to remind them that they had a duty to support research. Where sites had not issued permission but had received an eligible patient, we worked closely with regional CRNs to expedite approvals in order that consent to follow-up could be requested. Eventually all 95 sites agreed to support the study.

We made early contact with the NIHR CRNs for the regions covered by the collaborating ambulance services and utilised the support they provided to good effect [17]. The assistance provided by the CRNs when communicating with hospitals in their area was helpful, and intelligence regarding local resources and capacity was used in planning. We found some inconsistency in terms of CRN support for AIRWAYS-2, and this had a direct bearing on the time taken to secure local approvals. In the three regions where good support was in place we experienced minimal resistance to setting up the study, whereas in the one region where the local CRN was not fully supportive the timelines for approval were extended, and more time and effort was needed to gain agreement from the hospitals.

The electronic system was crucial when managing sites. In most trials with many hospitals taking part, a staggered approach to site start-up is used. In AIRWAYS-2 this was not possible since once patient enrolment began in an ambulance service, there was no way to predict which hospitals served by that ambulance service would receive a patient; approvals and training of staff at all 95 hospitals needed to be completed before patient enrolment commenced. Had this not been possible, there was a risk that participants would not be informed about the trial in a timely manner, and the opportunity to approach them and obtain consent for continued study involvement and data collection from those patients that survived to hospital discharge would be lost. The electronic study management and online training systems significantly reduced the time and resources required to deliver training to staff, and enabled the trial to operate virtually paper free throughout.

## Conclusion

EMS are increasingly research active. Incidents attended by EMS providers (paramedics) can be life-threatening emergencies, and this adds complexity to the design and implementation of research in this setting. Early and comprehensive engagement with key stakeholders is essential in the set-up of collaborative trials. Strategies to identify risks and practical problems before a study opens to recruitment can effectively mitigate against these, and a study-wide communication strategy can be utilised effectively.

The AIRWAYS-2 trial describes a methodological approach to successful and ethical research in the most severely ill and injured patients who lack capacity and require immediate life-saving treatment. The key features of study design and implementation described here can be used to support deliverable research that will lead to improved treatment and outcomes in this and similar patient groups.

## Additional files

Additional file 1: The electronic case report form (eCRF). (PDF 290 kb)

Additional file 2: Contributorship statement. (DOCX 16 kb)

## Abbreviations

CAG: Confidentiality Advisory Group
CRN: Clinical Research Network
eCRF: Electronic case report form
EMS: Emergency medical service
HTA: Health Technology Assessment
LC: Local collaborator
NIHR: National Institute for Health Research Clinical Research Network
OHCA: Out-of-hospital cardiac arrest
PI: Principal Investigator
PPI: Patient public involvement
RCT: Randomised controlled trial
SSI: Site-specific information

## References

[1] Taylor J, Black S, Brett SJ, Kirby K, Nolan JP, Reeves BC (2016). Design and implementation of the AIRWAYS-2 trial: a multi-centre cluster randomised controlled trial of the clinical and cost effectiveness of the i-gel supraglottic airway device versus tracheal intubation in the initial airway management of out of hospital cardiac arrest. Resuscitation.

[2] Benger JR, Kirby K, Black S, Brett SJ, Clout M, Lazaroo MJ, Nolan JP, Reeves BC, Robinson M, Scott LJ, Smartt H (2018). Effect of a strategy of a supraglottic airway device vs tracheal intubation during out-of-hospital cardiac arrest on functional outcome: the AIRWAYS-2 randomized clinical trial. JAMA.

[3] Chamberlain D, Cummins RO, Abramson N, Allen M, Baskett P, Becker L, et al. Recommended guidelines for uniform reporting of data from out-of-hospital cardiac arrest: the ‘Utstein style’. Resuscitation. 22(1):1–26. 10.1016/0300-9572(91)90061-3.

[4] Hawkes C, Booth S, Ji C, Brace-McDonnell SJ, Whittington A, Mapstone J (2017). Epidemiology and outcomes from out-of-hospital cardiac arrests in England. Resuscitation.

[5] Nichol G, Brown SP, Perkins GD, Kim F (2016). What change in outcomes after cardiac arrest is necessary to change practice? Results of an international survey. Resuscitation.

[6] Venkataraman A, Anderson P, Bierens J, et al. Prehospital research: an introduction: Falck Foundation; 2014. http://prehospitalresearch.eu/?p=3158. [Accessed 1 Dec 2017]

[7] Turner J, Nicholl J, Webber L, Cox H, Dixon S, Yates D (2000). A randomised controlled trial of prehospital intravenous fluid replacement therapy in serious trauma. Health Technol Assess (Winch Eng).

[8] Benger J, Coates D, Davies S, Greenwood R, Nolan J, Rhys M (2016). Randomised comparison of the effectiveness of the laryngeal mask airway supreme, i-gel and current practice in the initial airway management of out of hospital cardiac arrest: a feasibility study. Br J Anaesth.

[9] NHS England ambulance quality indicators. https://www.england.nhs.uk/statistics/statistical-work-areas/ambulance-quality-indicators/ Accessed 10 Jan 2018.

[10] Jansen TC, Kompanje EJO, Druml C, Menon DK, Wiedermann CJ, Bakker J (2007). Deferred consent in emergency intensive care research: what if the patient dies early? Use the data or not?. Intensive Care Med.

[11] Whitesides LW, Baren JM, Biros MH, Fleischman RJ, Govindarajan PR, Jones EB, Pancioli AM, Pentz RD, Scicluna VM, Wright DW, Dickert NW (2017). Impact of individual clinical outcomes on trial participants’ perspectives on enrollment in emergency research without consent. Clinical Trials.

[12] Woolfall K, Young B, Frith L, Appleton R, Iyer A, Messahel S, Hickey H, Gamble C (2014). Doing challenging research studies in a patient-centred way: a qualitative study to inform a randomised controlled trial in the paediatric emergency care setting. BMJ Open.

[13] Kearney A, McKay A, Hickey H, Balabanova S, Marson AG, Gamble C, Williamson P (2014). Opening research sites in multicentre clinical trials within the UK: a detailed analysis of delays. BMJ Open.

[14] Model agreement for non-commercial research in the Health Service. https://www.ukcrc.org/regulation-governance/model-agreements/. Accessed 6 Dec 2017.

[15] NIHR site identification. https://www.nihr.ac.uk/funding-and-support/study-support-service/site-identification/. Accessed 31 Jan 2018.

[16] NIHR in your area, Local Clinical Research Networks. https://www.nihr.ac.uk/nihr-in-your-area/local-clinical-research-networks.htm. Accessed 31 Jan 2018.

[17] Lecouturier J, Rodgers H, Ford GA, Rapley T, Stobbart L, Louw SJ, Murtagh MJ (2008). Clinical research without consent in adults in the emergency setting: a review of patient and public views. BMC Med Ethics.

[18] The Medicines for Human Use (Clinical Trials) Amendment (No.2) Regulations 2006. http://www.legislation.gov.uk/uksi/2006/2984/contents/made.

[19] Hackshaw A, Farrant H, Bulley S, Seckl MJ, Ledermann JA (2008). Setting up non-commercial clinical trials takes too long in the UK: findings from a prospective study. J R Soc Med.

[20] Kielmann T, Tierney A, Porteous R, Huby G, Sheikh A, Pinnock H (2007). The Department of Health’s research governance framework remains an impediment to multi-centre studies: findings from a national descriptive study. J R Soc Med.

[21] Thompson AG, France EF (2010). One stop or full stop? The continuing challenges for researchers despite the new streamlined NHS research governance process. BMC Health Serv Res.

[22] Snooks H, Hutchings H, Seagrove A, Stewart-Brown S, Williams J, Russell I (2012). Bureaucracy stifles medical research in Britain: a tale of three trials. BMC Med Res Methodol.

