# Supplementary material for: Design and implementation of a large and complex trial in emergency medical services
Source: Trials. 2019 Feb 8;20:108. doi: 10.1186/s13063-019-3203-0 (PMC6368693; doi:10.1186/s13063-019-3203-0)
Supplement: Supplementary file 1 — The electronic case report form (eCRF). (PDF 290 kb) [file 13063_2019_3203_MOESM1_ESM.pdf]

Form A - COMPLETE FOR ALL OCHAs

A (1)

Incident details

Incident ID: 1602010975

Incident date: [redacted]

Incident time: 17:09

Identifying patients

Identified On: CAD: Yes

Patient identified in ambulance service data: Yes

Paramedic CRF received: Yes

Resuscitation details

Was resuscitation attempted? Yes

If resuscitation was not attempted, reason(s) :-

Massive cranial and cerebral destruction	No
Hemicorporectomy	No
Massive truncal injury imcompatible with life	No
Decomposition/ putrefaction	No
Incineration	No
Hypostasis	No
Rigor mortis	No
Patient's wishes/ DNAR	No
Death expected due to terminal illness	No
Efforts would be futile	No
Submersion >1hour	No
Other	No

Details:

Paramedic details

Was incident attended by an AIRWAYS-2 paramedic: Yes

If YES, AIRWAYS-2 paramedic ID (of first A2 paramedic on scene) [redacted]

If the paramedic who treated or enrolled the patient in the trial was not the first A2 paramedic on scene, please provide reasons and details:

Delete Save

Form B - COMPLETE FOR OHCAs WITH RESUSCITATION ATTEMPTED

B (1)

Patient Details

Estimated age

or

Date of birth [redacted]

Gender: Female

Initial cardiac arrest details

Presenting rhythm	VF
Was the arrest witnessed?	Yes
If yes, who was it witnessed by?	EMS
Was there bystander CPR?	No

First crew details

Date of first crew arrival: 01/02/2016

Time of first crew arrival: 17:26

Delete Save

Form C - COMPLETE FOR OHCAs WITH RESUSCITATION ATTEMPTED AND ATTENDED BY AIRWAYS-2 PARAMEDIC

C (1)

Patient eligibilty

Did the patient meet eligibility criteria?	Yes
If NO, why?	
- Aged < 18 years	No
- Traumatic cardiac arrest	No
- In-hospital cardiac arrest	No
- Airways paramedic not 1st/2nd at patient's side	No
- Advanced airway management inserted by another HCPC registered paramedic, doctor or nurse already in place when AIRWAYS-2 paramedic arrives at patient's side (when the first paramedic to arrive is not participating in AIRWAYS-2)	No
- Resuscitation was not commenced or continued by ambulance staff or responder	No
- Patient detained by HMP	No
- Previously recruited to trial	No
- Patient previously expressed wishes not to participate	No
- Mouth opening < 2cm	No
- Patient did not have an OHCA	No

ENROLMENT DETAILS

If patient was eligible (or believed to be eligible at the time of airway management), were they consciously enrolled in AIRWAYS-2?	Yes
If NO, reason	-
If NO, reason, other	

Delete Save

Form D - COMPLETE FOR AIRWAYS-2 PATIENTS

D (1)

ED ADMISSION DETAILS

Patient Name	[redacted] (?)
Was patient admitted to ED?	Yes
if YES:	
Which hospital?	SW17 - Southampton General
- If Other hosptial, please specify	
Date of arrival	01/02/2016
Approx time of arrival	19:02

Delete Save

Form G - Patient details

G (1)

Patient details

Please check the patient details and update as required

Name	[redacted]
Gender	Female
Which hospital?	SW17 - Southampton General
If other, details	
Date admitted	01/02/2016
Time admitted	19:02
Approximate age (for identification purposes)	55

ICU (OR EQUIVELENT LEVEL OF CARE) DETAILS

NHS Number	[redacted]	Date of birth	[redacted]
**Did patient survive to ICU admission?**	Yes		
If No, date/time of death			
If Yes, date/time of arrival in ICU		01/02/2016 19:46	
**Did patient survive to ICU discharge?**	Yes		
If No, date/time of death			
If Yes, date/time of ICU discharge		01/02/2016 19:46	

Hospital transfer details

Was patient transferred to another hospital prior to approach?	No
If YES, Which hospital?	-
- If Other hosptial, please specify	

Have there been any additional ward movements (post ICU discharge) prior to transfer? -

If yes, please complete below

Admission / transfer date	Admission / transfer time	Level of care for this admission	Destination code (i.e. level of care the patient will move to on leaving this admission)
1			

Date of hospital transfer		Time of hospital transfer	

PATIENT CAPACITY (only required if patient survived to ICU discharge)

Does patient have capacity to consent? Yes

Delete Save

PATIENT SCREENING

H1 (1)

PATIENT APPROACH: COMPLETE FOR ALL PATIENTS

Was the patient approached in hospital?	Yes
If NO, please provide reason	*Research team not notified of patient until 5 months after dis*
If YES, date of approach	*30/08/2016*
If the patient was approached in hospital were they given a PIL?	Yes
If NO, please provide reason	
If the patient was not approached in hospital, was study information (including PIL) sent in the post?	-
If NO, please provide reason	
If YES:	
Date information sent	
Did the patient respond?	-

PATIENT CONSENT: COMPLETE IF PIL GIVEN / PIL SENT AND PATIENT RESPONDED

Which consent option did the patient select?	*A (Active follow up)*	Date of consent	*30/08/2016*
		Please upload consent form to the database	SW03481_Airways pt consent form [redacted].pdf ☐ Delete<br>Browse...<br>**Upload Status:**

Name of person completing form:	*Clare Bolger*		
Signature of person completing form completed?:	Yes	Date completed	*30/08/2016*

Delete Save

IN HOSPITAL DETAILS

H3 (1)

COMPLETE THIS FORM FOR PATIENTS WHO HAVE CONSENTED UNDER OPTIONS A OR B

Patient address		GP address	
Line 1	[redacted]	Line 1	[redacted]
Line 2		Line 2	
Town / City		Town / City	
County		County	
Postcode	[redacted]	Postcode	[redacted]
Patient phone number	[redacted]	GP name	[redacted]
Can answer machine messages be left?	Yes	GP phone number	[redacted]
Patient email address		Has GP letter been downloaded and filed in patient notes?	Yes

Patient's preferred mode of contact for follow up questionnaires (tick all that apply)

Post	Yes
Phone	No
Email/web	No

WARD MOVEMENTS

Admission / transfer date	Admission / transfer time	Level of care for this admission	Destination code (i.e. level of care the patient will move to on leaving this admission)	
01/02/2016	19:47	Level 3 (e.g. ITU)	2 (Level 2)	1.
01/02/2016	21:30	Level 2 (e.g. HDU)	1 (Level 1)	2.
03/02/2016	15:53	Level 1 (e.g. General)	4 (Hospital discharge)	3.

1

* Note:- date/time of first admission should match ICU discharge date/time on Form G/G2

DISCHARGE DETAILS

Did patient survive to hospital discharge? Yes

If NO, date/time of death

If Yes:

Date/time of discharge 09/02/2016 18:00

Discharge destination Home

If other hospital, please select hospital name -

If other, details

Name of person completing form: Karen Salmon/ katie sweet

Signature of person completing form completed?: Yes

Date completed 02/09/2016

Delete Save
