# Supplementary material for: Design and implementation of a large and complex trial in emergency medical services
Source: Trials. 2019 Feb 8;20:108. doi: 10.1186/s13063-019-3203-0 (PMC6368693; doi:10.1186/s13063-019-3203-0)
Supplement: Supplementary file 2 — Contributorship statement. (DOCX 16 kb) [file 13063_2019_3203_MOESM2_ESM.docx]

**Additional file 2: Contributorship Statement**

The AIRWAYS-2 study team, and the contribution made by each individual, are described in the following statement. The Chief Investigator and study guarantor is Jonathan Benger. The research question was developed and the study was designed by Jonathan Benger, Sarah Black, Stephen Brett, Kim Kirby, Jerry Nolan, Barnaby Reeves, Megan Rhys, Maria Robinson, Chris Rogers, Lauren Scott, Adrian South, Elizabeth Stokes, Jodi Taylor, Matthew Thomas, Sarah Voss and Sarah Wordsworth. The study protocol was implemented by Jonathan Benger, Sarah Black, Stephen Brett, Rachel Brophy, Madeleine Clout, Kim Kirby, Jenny Lamb, Michelle Lazaroo, Jerry Nolan, Barnaby Reeves, Maria Robinson, Chris Rogers, Lauren Scott, Helena Smartt, Adrian South, Elizabeth Stokes, Jodi Taylor, Matthew Thomas, Sarah Voss, Abby Willcox and Sarah Wordsworth. Financial management was provided by Adam Wallis, Tom Hill and Tony West. Data collection in the four participating ambulance trusts was coordinated by research paramedics: Jonathan Green (South Western); Helen Hall (East of England); Kim Kirby (central study team); Richard Pilbery (Yorkshire); Greg Whitley (East Midlands). Research support and governance in the four participating ambulance trusts was provided by: Maria Robinson (South Western); Theresa Foster (East of England); Jane Shewan (Yorkshire); Anne Spaight (East Midlands). Principal investigators in each of the four participating Ambulance Trusts were: Adrian South (South Western); Marcus Bailey (East of England); Steven Dykes (Yorkshire); Niro Siriwardena (East Midlands). The regional research nurses who coordinated and supported data collection in all receiving hospitals were: Lisa Grimmer (Bristol Royal Infirmary); Katie Sweet (Bristol Royal Infirmary); Rosalyn Squire (Derriford Hospital, Plymouth); Prematie Andreou (Leicester Royal Infirmary); Lucy Ryan (Nottingham University Hospital); Sara Jones (Addenbrookes Hospital); Helen Foot (Northern General Hospital, Sheffield). The independent members of the Trial Steering Committee were: Simon Gates (Chair), Charles Deakin, Keith Douglas, Margaret Douglas, Gavin Perkins and Jasmeet Soar. The independent members of the Data Monitoring Committee were: Gordon Taylor (Chair), Richard Lyon, Andrew Newton, Tom Quinn and Helen Snooks.
